# Synthesis of Mo_2_C and W_2_C Nanoparticle Electrocatalysts for the Efficient Hydrogen Evolution Reaction in Alkali and Acid Electrolytes

**DOI:** 10.3389/fchem.2019.00716

**Published:** 2019-10-25

**Authors:** Sajjad Hussain, Dhanasekaran Vikraman, Asad Feroze, Wooseok Song, Ki-Seok An, Hyun-Seok Kim, Seung-Hyun Chun, Jongwan Jung

**Affiliations:** ^1^Graphene Research Institute, Sejong University, Seoul, South Korea; ^2^Department of Nano and Advanced Materials Engineering, Sejong University, Seoul, South Korea; ^3^Division of Electronics and Electrical Engineering, Dongguk University-Seoul, Seoul, South Korea; ^4^Department of Physics, Sejong University, Seoul, South Korea; ^5^Thin Film Materials Research Center, Korea Research Institute of Chemical Technology, Daejeon, South Korea

**Keywords:** Mo_2_C, W_2_C, nanoparticle, HER, electrocatalyst

## Abstract

The synthesis of low cost, high efficacy, and durable hydrogen evolution electrocatalysts from the non-noble metal group is a major challenge. Herein, we establish a simple and inexpensive chemical reduction method for producing molybdenum carbide (Mo_2_C) and tungsten carbide (W_2_C) nanoparticles that are efficient electrocatalysts in alkali and acid electrolytes for hydrogen evolution reactions (HER). Mo_2_C exhibits outstanding electrocatalytic behavior with an overpotential of −134 mV in acid medium and of −116 mV in alkaline medium, while W_2_C nanoparticles require an overpotential of −173 mV in acidic medium and −130 mV in alkaline medium to attain a current density of 10 mA cm^−2^. The observed results prove the capability of high- and low-pH active electrocatalysts of Mo_2_C and W_2_C nanoparticles to be efficient systems for hydrogen production through HER water electrolysis.

## Introduction

A direct and effective route to clean and renewable hydrogen (H_2_) production by water splitting requires a robust catalyst to ensure sustainable efficiency (Vikraman et al., 2017). Platinum (Pt)-based systems are recognized as highly energetic HER catalysts that boast various pH tolerances and almost zero overpotential, but their high cost, originating from the scarcity of platinum, severely hinders their extensive use (Jacobsson et al., 2013; Peng et al., 2014). To reduce the use of Pt, intensive research has been conducted to prepare low-cost, highly active, and electrochemically stable HER electrocatalysts comprised of abundant elements as an alternative (Vikraman et al., 2018; Hussain et al., 2019a). Numerous types of non-scarce transition metal compounds, such as layered transition metal dichalcogenides (LTMDs) (Laursen et al., 2012; Seok et al., 2017; Hussain et al., 2018), phosphides (Lv and Wang, 2017; Pei et al., 2018), transition metal carbides (TMC) (Huang et al., 2016; Shi et al., 2017), and their composites (Ren et al., 2018; Vikraman et al., 2019b) have been shown to produce excellent HER activities because of their unique electronic structures. Various molybdenum selenide (MoSe_2_)-based materials, in particular, have been reported as being suited for replacing Pt in HER electrocatalysts (Ren et al., 2018; Hussain et al., 2019a; Vikraman et al., 2019a). In addition, attaching Mo_2_C and W_2_C to a carbon matrix has been shown to produce high-rate charge transfer properties during HER and to alleviate surface aggregation (Pan et al., 2014; Youn et al., 2014; Wu et al., 2016). Previous reports suggested that the HER electrocatalytic performance of Mo_2_C- and W_2_C-based catalysts mainly results from the morphology (Ang et al., 2016; Ishii et al., 2016; Peng et al., 2017), crystalline phases (Wan et al., 2014; Lin et al., 2017), and composition (Yu et al., 2018; Zhang et al., 2018) of the catalysts and the synthetic protocol. However, the critical challenge is to design and develop carbide-based catalysts with comparable catalytic performance to Pt for practical applications. Until now, carbide-based materials have shown inferior catalytic properties due to their poor activities (Wu et al., 2016; Yu et al., 2018; Zhang et al., 2018). An earlier study reported the HER behavior of commercially available Mo_2_C meshes in both basic and acidic solutions, showing a comparatively high overpotential of −190~-230 mV at a cathodic current of 10 mAcm^−2^ (Vrubel and Hu, 2012). Performance has since been upgraded by tuning its nanocrystal size (Ma R. et al., 2015; Chen et al., 2016). Hence, Mo_2_C and W_2_C nanoparticles with high catalytic activity and robustness remain open for future research.

Various methods have been employed to prepare TMC-based catalysts (Wu et al., 2016; Zhu et al., 2016; An and Xu, 2019). Gong et al. obtained WC_x_/MoC_x_-based electrocatalysts by carbonizing W/WO_3_ or Mo/MoO_3_ under the flow of carbon precursors (C_2_H_6_, CH_4_, or CO, and H_2_), but very low surface areas were obtained (Gong et al., 2016). Tantalum carbide nanocrystals have been prepared via a refined Cl_2_-assisted “micro-cutting-fragmentation” approach and produced an overpotential of ~-146 mV@10 mA cm^−2^ for HER (Kou et al., 2017). Different methodologies have been used to prepare Mo_2_C and its hybrids for HER applications (Pu et al., 2016; Kou et al., 2018; Liang et al., 2019). Tungsten carbides can exist in different phases, such as WC, metastable W_2_C, and WC_1−x_, but during the last decade, most researchers have exclusively focused on the WC phase instead of the W_2_C phase (Neylon et al., 1999; Ishii et al., 2016). W/WC synthesized by Kou et al. (2019) showed an overpotential of 159 mV @ 10 mA cm^−2^. As per experimental and theoretical calculations, the W_2_C phase exhibits more HER active catalytic properties than does WC, with a low negative Gibbs free energy (ΔGH) for hydrogen adsorption and high Fermi level electronic density of states (DOS) (Colton et al., 1975; Gong et al., 2016). β-phase Mo_2_C and the W_2_C phase, in particular, have been validated as effective HER electrocatalysts despite their bulky natures, and their activity could be further increased by engineering with appropriate nanosize structures (Huang et al., 2016).

Herein, we have successfully synthesized Mo_2_C and W_2_C nanoparticles for HER application via a simple and economical chemical reduction method. The electrochemical results indicate that Mo_2_C nanoparticles exhibit superior HER electrocatalytic behavior over W_2_C in both alkaline and acid solutions, with small Tafel slopes and distinct solidity. We believe that our present investigation provides effective strategies for designing and preparing the new nanostructured TMC-based HER catalysts.

## Results and Discussion

**Figure 1:**
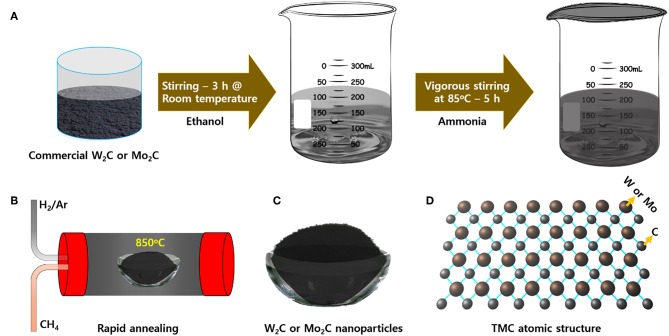
Schematic representation of the **(A)** chemically reduction, **(B)** thermal annealing at 850°C, **(C)** prepared powders, and **(D)** atomic structure of Mo_2_C and W_2_C nanoparticles.

**Figure 2:**
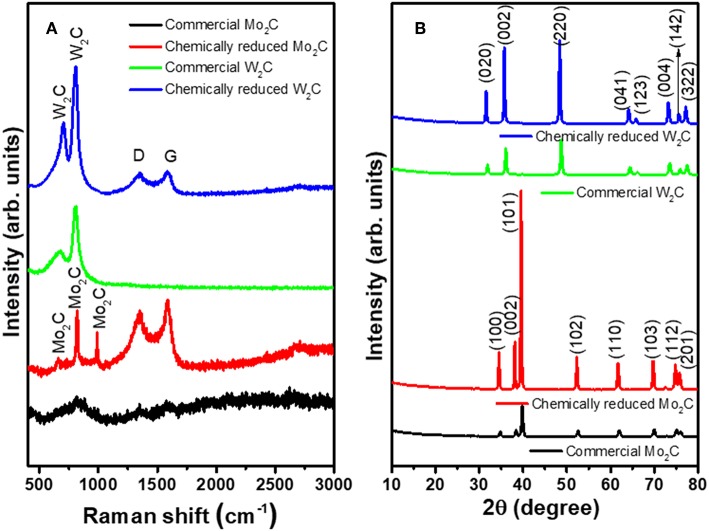
**(A)** XRD patterns and **(B)** Raman spectra of commercial Mo_2_C and W_2_C and chemically reduced Mo_2_C and W_2_C.

The chemically reduced synthesis scheme and the atomic structures of the Mo_2_C and W_2_C nanoparticles are pictorially represented in Figure 1. Raman characterization was performed for structural confirmation of commercial and chemically reduced Mo_2_C and W_2_C nanoparticles. Figure 2A shows the Raman spectra of commercial Mo_2_C and W_2_C and chemically reduced Mo_2_C and W_2_C. The commercial Mo_2_C revealed unclear peaks, whereas the chemically reduced Mo_2_C produced distinctive peaks at 661, 818, and 990 cm^−1^, credited to β-Mo_2_C (Hussain et al., 2019b). In addition, the G band was positioned at 1,582 cm^−1^ and the D band was positioned at 1,349 cm^−1^, which corresponds to the sp^2−^ bonded carbon atoms and disarranged graphite carbon, respectively (Ma F. et al., 2015). The intensity ratio of the D-band to the G-band (i.e., I_D_/I_G_) is 0.85 for Mo_2_C, comparable to long-time carbonized graphene layers with high conductivity, which may facilitate charge transfer during HER (Li et al., 2016; Lv et al., 2017; Wang et al., 2017). For commercial and chemically reduced W_2_C, the strong peaks observed at 693 and 807 cm^−1^ originate from the stretching vibration of W-C (Yan et al., 2017). The D and G bands were exhibited at 1,351 and 1,583 cm^−1^ for the chemically reduced W_2_C (Yan et al., 2017; Zhang et al., 2018). The observed results are in good agreement with previous reports (Yan et al., 2017; Hussain et al., 2019a).

X-ray diffraction (XRD) was investigated to reveal the material structure of the commercial and chemical Mo_2_C and W_2_C nanoparticles (Figure 2B). For Mo_2_C, the XRD patterns revealed reflections at 34.5, 38.1, 39.5, 52.3, 61.7, 69.6, 74.7, and 75.7°, which were indexed to the (100), (002), (101), (102), (110), (103), (112), and (201) planes of β-Mo_2_C (JCPDS: 35-0787), which is the most active phase of Mo_2_C for HER. In the case of W_2_C, the XRD patterns produced peaks at 31.5, 35.1, 48.8, 64.1, 65.8, 73.2, 75.6, and 77.1° due to the (020), (002), (220), (041), (123), (004), (142), and (322) lattice planes, respectively (JCPDS: 89-2371). Compared with commercial samples, the chemically reduced samples showed higher-intensity peaks, which can be attributed to the enriched crystallinity in the nanoparticles compared to in bulk (Hussain et al., 2019a). No discernible peaks in either the Mo_2_C or W_2_C samples could be assigned to Mo/W, carbide, or other Mo/W non-stoichiometric phases and impurities, indicating the capability of the synthetic method to prepare effective nanostructures from bulk.

**Figure 3:**
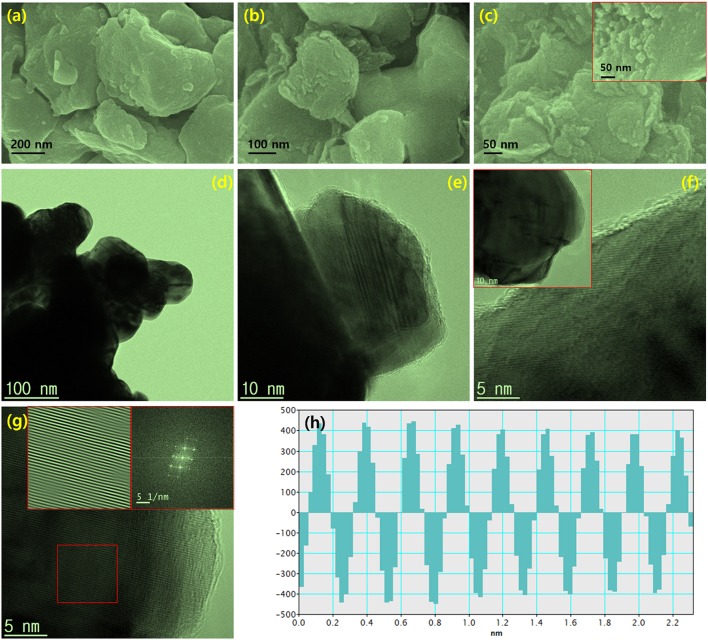
**(a–c)** FESEM images at different magnifications for chemically reduced Mo_2_C nanoparticles, **(d–h)** TEM micrographs of Mo_2_C nanoparticles, **(d–f)** Different magnifications of TEM images of dispersed nanosheet structures, **(g)** High-resolution TEM image with the typical fingerprint-type layered structure, with insets of FFT and inverse FFT patterns obtained via point mask mode, and **(h)** intensity profile.

Morphology and microstructure modifications were inspected using field emission scanning electron microscopy (FESEM) and transmission electron microscopy (TEM). Figures 3a–c show FESEM images for reduced Mo_2_C nanoparticles. The observed images clearly indicated that larger sized grains were constructed through the accumulation of spherically shaped nanoparticles. The composition of reduced Mo_2_C nanoparticles was examined by energy dispersive spectroscopy (EDS), as shown in Figure S1. The observed composition of the chemically reduced sample is in good agreement with the claim of Mo_2_C formation, and EDS mapping images for the nanoparticles are provided in Figure S2. TEM images at different magnifications of Mo_2_C nanoparticles are provided in Figures 3d–g. The large grains are clearly seen in the lower-magnification TEM image (Figure 3d), and the typical 2D layered structures are shown in the higher-magnification images. A high-resolution TEM image showing a fingerprint-like structure is shown in Figure 3g, and its fast Fourier transform (FFT) and inverse FFTs are inserted. The intensity profile extracted proved the existence of (101) lattice plane spacing (0.228 nm) between the fringes, as shown in Figure 3h. Hence, the microscopic images clearly proved the formation of Mo_2_C nanoparticles.

**Figure 4:**
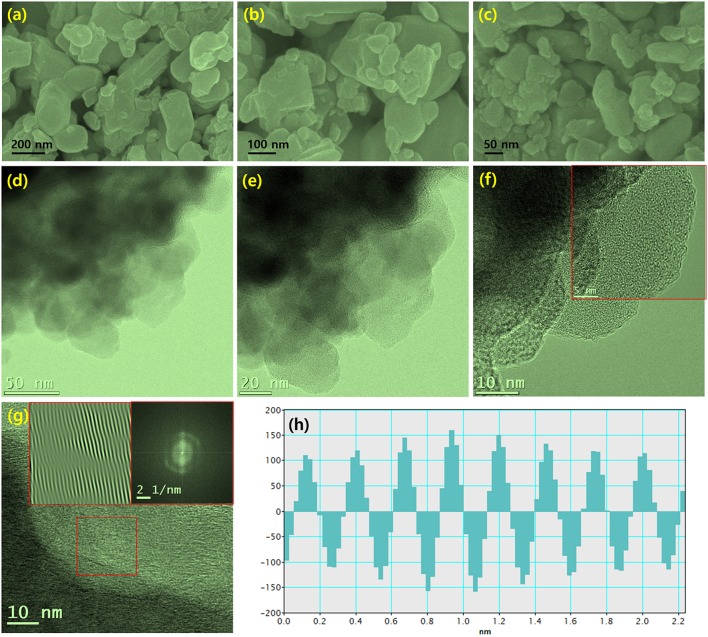
**(a–c)** FESEM images of different magnification for chemically reduced W_2_C nanoparticles, **(d–h)** TEM micrographs of W_2_C nanoparticles **(d–e)** Different magnifications of TEM images of accumulated nanosheet structures, **(f,g)** high-resolution TEM images of interconnected layers with a fingerprint structure, with an inset showing the inverse FFT pattern obtained via point mask mode, and **(h)** intensity profile.

Figure 4 shows FESEM and TEM images of chemically reduced W_2_C nanoparticles. The nanoparticles consist of inhomogeneous grains (Figures 4a–c). The EDS spectrum confirmed the existence of W_2_C nanoparticles (Figure S3), and their mapping images further supported the claim of W_2_C nanoparticle formation (Figure S4). The TEM images provided valuable insights regarding the W_2_C nanoparticles (Figures 4d–g). The irregularly sized nanograins were clearly seen in the low- and higher-magnification TEM images (Figures 4f,g). The FFT image showed the (002) W_2_C plane (0.26 nm spacing) (Figure 4h). The observed results are well-correlated with the XRD results.

**Figure 5:**
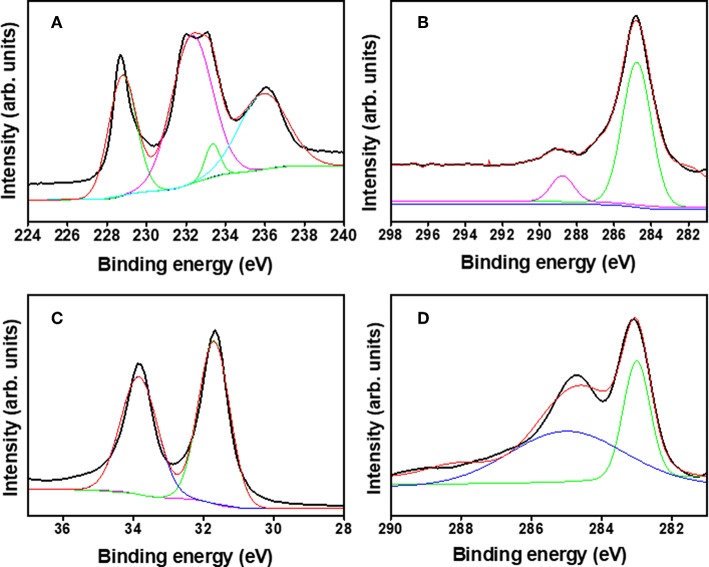
X-ray photoemission spectra: **(A)** Mo 3d, **(B)** C 1s binding energy spectra of Mo_2_C nanoparticles, **(C)** W 4f, and **(D)** C 1s binding energy spectra of W_2_C nanoparticles.

An X-ray photoelectron spectroscopy (XPS) study was conducted to validate the composition of reduced Mo_2_C and W_2_C. The XPS survey spectra revealed the occurrence of Mo_2_C (C and Mo)- and W_2_C (C and W)-based elements, as shown in Figures S5A,B. Figure 5A shows the de-convoluted high-resolution region for Mo 3d, with the Mo 3d_5/2_ and Mo 3d_3/2_ peaks at 228.6 and 231.9 eV, which reveals the carbidic Mo phase. The peaks at 233.3 and 235.9 eV are from molybdenum oxide (Mo^6+^), indicating surface oxidation in an air environment (Cui et al., 2014; Tang et al., 2015; Huang et al., 2016; Fan et al., 2018; Hussain et al., 2019a). The C 1s spectrum (Figure 5B) at a 284.8 eV binding energy is characteristic of the sp^2^ carbon relation in Mo_2_C, whereas a satellite peak emerges at 288.8 eV due to C–O bonding (Wu et al., 2016). Figure 5C shows the high-resolution W 4f XPS spectrum from chemically reduced W_2_C. The deconvoluted peaks revealed at 31.64 and 33.81 eV were credited to 4f_7/2_ and 4f_5/2_, respectively, for the W 4f atom. The high-resolution C 1s spectrum (Figure 5D) of W_2_C exposed the sp^2^ graphitic carbon peak at 283.02 eV and C = O peak at 285.1 eV (Berglund et al., 2014; Ko et al., 2017). The observed results confirmed the formation of Mo_2_C and W_2_C, which is in good agreement with the earlier literature (Ma F. et al., 2015; Yan et al., 2017). The surface area modifications were measured by the nitrogen (N_2_) adsorption/desorption isotherms for chemically reduced Mo_2_C and W_2_C nanoparticles via the Brunauer Emmett Teller (BET) method (Figures S6A,B). Surface area of 0.91 and 1.75 m^2^·g^−1^ were observed for the chemically reduced Mo_2_C and W_2_C, respectively, compared with the reported values of their commercial samples (Gao et al., 2014; Hussain et al., 2019a). In addition, the pore diameter vs. pore volume profile (Figure S6B) shows the mesoporous nature of chemically reduced Mo_2_C and W_2_C, with pore diameters of 14.9 and 21.4 nm and pore volumes of 0.003 and 0.009 cm^3^·g^−1^, respectively.

**Figure 6:**
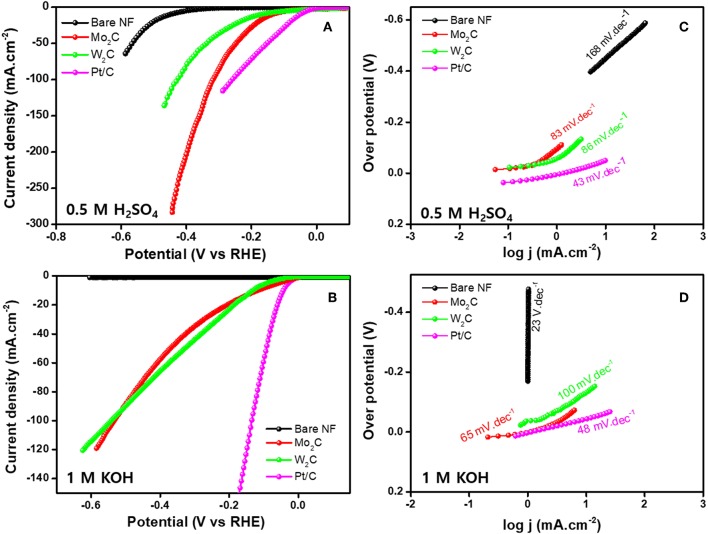
HER performance of bare NF, Pt/C, Mo_2_C, and W_2_C nanoparticle-decorated NF. **(A,C)** LSV profiles and their Tafel plots in 0.5 M H_2_SO_4_ and **(B,D)** LSV profiles and their Tafel plots in 1.0 M KOH.

The HER electrocatalytic activities of Mo_2_C- and W_2_C-coated nickel foam (NF) electrodes were analyzed by linear sweep voltammogram (LSV) in 1 M KOH and 0.5 M H_2_SO_4_ electrolyte solutions (scan rate of 10 mV s^−1^, three-electrode setup). For the evaluation, a commercial Pt/C was used as an electrode, and the results were as follows. Figure 6A shows the LSV curves of commercial Pt/C and reduced Mo_2_C and W_2_C catalysts in a 0.5 M H_2_SO_4_ solution. As anticipated, the commercial Pt/C catalyst showed the highest HER activity, which had an overpotential of ~-49 mV. For bare NF, Mo_2_C, and W_2_C, overpotentials of −423, −134, and −170 mV were needed to achieve 10 mAcm^−2^ in acidic electrolyte, respectively, lower than those of the commercial samples (Vrubel and Hu, 2012; Chen et al., 2013). The overpotentials of the chemically reduced Mo_2_C and W_2_C were much closer to the recently reported values for carbide-based materials in acid medium, such as Mo_2_C encapsulated by nitrogen and phosphorus codoped carbon shells ($\eta_{10\text{mA}/\text{c}{\text{m}}^{2}}$@-260 mV) (Li et al., 2016), Mo_2_C nanoparticles embedded in chain-like Ketjenblack carbon ($\eta_{10\text{mA}/\text{c}{\text{m}}^{2}}$@-221 ~263 mV) (Wang et al., 2017), porous MoC_x_ nano octahedrons ($\eta_{10\text{mA}/\text{c}{\text{m}}^{2}}$@-142 mV) (Wu et al., 2015), 3D porous scaffold-like Mo_2_C/C nanosheet hybrids ($\eta_{10\text{mA}/\text{c}{\text{m}}^{2}}$@-233 mV) (Wang et al., 2018), reduced graphene oxide-based Mo_2_C composites ($\eta_{10\text{mA}/\text{c}{\text{m}}^{2}}$@-206 mV) (Ojha et al., 2016), Mo_2_C nanoparticle-decorated graphitic carbon sheets ($\eta_{10\text{mA}/\text{c}{\text{m}}^{2}}$@-200~210 mV) (Cui et al., 2014), Mo_2_C particles embedded in a sulfur and nitrogen codoped mesoporous carbon matrix ($\eta_{10\text{mA}/\text{c}{\text{m}}^{2}}$@-146 mV) (An et al., 2017), and Mo_2_C NCs on vertically aligned graphene ($\eta_{10\text{mA}/\text{c}{\text{m}}^{2}}$@-152 mV) (Fan et al., 2017). The electrocatalytic properties of Pt/C, Mo_2_C, and W_2_C catalysts were appraised in a 1 M KOH medium (Figure 6B). Similarly, the Pt/C, Mo_2_C, and W_2_C electrocatalysts produced −48, −116, and −130 mV overpotentials to drive the 10 mAcm^−2^ reaction in an alkaline electrolyte. The bare NF did not produce viable HER properties in a 1 M KOH medium. The observed results constitute a considerable advance over earlier results.

The Tafel slope signifies the characteristic activity acquired of an electrocatalyst and is derived using the Tafel equation for the HER. As shown in Figures 6C,D, Pt/C offers Tafel slopes of 43 and 48 mV.dec^−1^ for acidic and alkaline electrolytes, respectively, which is comparable with the reported results (Fan et al., 2018). The extracted slope values from the Tafel plots were 83 and 86 mV dec^−1^ for the acidic and 65 and 100 mV.dec^−1^ for the alkali for reduced Mo_2_C and W_2_C, respectively. These values indicate that the Volmer-Heyrovsky reaction step is obeyed for the whole HER process (Bockris and Potter, 1952; Yuan et al., 2018). Another important parameter, the exchange current density (j_0_), was extracted to evaluate HER activity by extrapolating the linear region of the Tafel plot. A chemically reduced Mo_2_C catalyst displays j_0_ values of 0.846 and 0.131 mA cm^−2^ in alkali and acidic media, respectively, while a chemically reduced W_2_C catalyst shows j_0_ values of 0.438 and 0.194 mA cm^−2^. The observed parameters are provided in Table 1. A detailed comparison of chemically reduced Mo_2_C and W_2_C HER parameters, with the reported carbide-based materials in alkaline and acidic media, are provided in Tables S1, S2, respectively.

**Table 1:** Comparison of the electrochemical parameters of different electrocatalysts.

**Electrolyte**	**Electrocatalysts**	**Tafel slope (mV·dec^**−1**^)**	**Overpotential (mV vs. RHE) @ 10 mA·cm^**−2**^**	**Exchange current density (j_**0**_, mA·cm^**−2**^)**
0.5 M H_2_SO_4_	Pt/C	43	−49	1.03
	Mo_2_C	83	−134	0.131
	W_2_C	86	−173	0.194
	Bare NF	168	−423	0.022
1 M KOH	Pt/C	48	−48	0.891
	Mo_2_C	65	−116	0.846
	W_2_C	100	−130	0.438
	Bare NF	–	–	–

The observed HER properties are credited to the reduced sizes of Mo_2_C and W_2_C nanostructures and their improved morphological properties in terms of edge sites.

High electrical conductivity allows for rapid ion/electron transfer between the active electrocatalyst edge sites and also provides a high interaction area between the electrolyte and the electrode, which in turn enhances the electrochemical performance. The charge transport mechanism was clarified by employing electrochemical impedance spectroscopy (EIS) studies to understand the HER mechanism of Mo_2_C and W_2_C nanoparticles at the interface between electrodes and electrolytes. The charge transfer properties with resistance (R_ct_) are exhibited for Pt/C in acidic and alkaline media as shown in Figures S7A,B. The lower R_ct_ values for chemically reduced Mo_2_C and W_2_C in acidic and alkaline media confirmed that they favor rapid electron transport in H_2_ evolution (Figures S7A,B). We estimated the electrochemically active surface area (ECSA) through cyclic voltammetry (CV), which was accomplished at different scan rates from 10 to 100 mVs^−1^ in the non-Faradaic regions for Mo_2_C and W_2_C, as shown in Figures S8A–D, respectively (Zhou et al., 2016; Hussain et al., 2019a; Vikraman et al., 2019a). The double-layer capacitance (C_dl_) was extracted from the fitted slope value of the current differences (Δj_a−c_) of the cathodic and anodic peaks of CV profiles at 0.24 V vs. RHE (Figure S8E). The extracted C_dl_ values were 2.13 and 1.42 mF.cm^−2^ in acid medium and 2.90 and 1.86 mF.cm^−2^ in alkaline medium for Mo_2_C and W_2_C, respectively. The ECSA values were assessed through a previously described procedure (Vikraman et al., 2019a) and were 60.8 and 40.6 cm^2^ in an acidic and 72.5 and 46.5 cm^2^ in a alkaline medium for Mo_2_C and W_2_C, respectively, indicating higher HER activity for the Mo_2_C system. We estimated the amount of surface active sites using the method reported by Fei et al. (2015). The turnover frequency (TOF) values of Mo_2_C and W_2_C were calculated in the acidic and alkaline media. The TOF values of Mo_2_C are 0.005 and 0.037 H_2_.s^−1^ at overpotentials of −134 and −116 mV, and those of W_2_C are 0.015 and 0.034 H_2_.s^−1^ at overpotentials of −173 and −130 mV in acidic and alkaline media, respectively. The observed TOF values are comparable with previous reports (Chen et al., 2011; Fei et al., 2015; Ma L. et al., 2015; Zhang et al., 2017).

**Figure 7:**
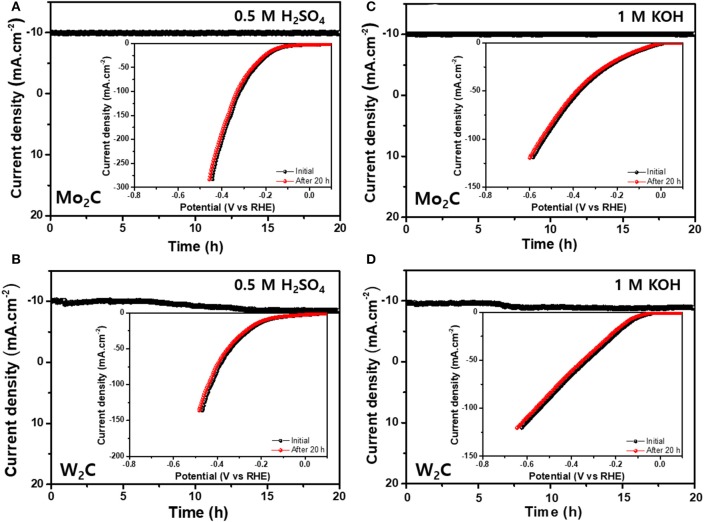
Long-term stability tests **(A,B)** chronoamperometric curves for Mo_2_C- and W_2_C-loaded NF electrocatalyst in 0.5 M H_2_SO_4_ (inset: corresponding LSV profiles for initial and after 20 h of HER performance) and **(C,D)** Chronoamperometric curves for Mo_2_C- and W_2_C-loaded NF electrocatalyst in 1.0 M KOH (inset: corresponding LSV profiles for initial and after 20 h of HER performance).

The robustness of the Mo_2_C and W_2_C catalysts was analyzed by carrying out chronoamperometric performances in 0.5 M H_2_SO_4_ and in 1 M KOH for 20 h; the results are shown in Figures 7A–D. The LSV profiles of Mo_2_C and W_2_C catalysts were examined after 20 h of HER operation (−0.6 to 0.15 V vs. RHE at a scan rate of 10 mV s^−1^) in alkaline and acid media (Figure 7). The polarization curves revealed the robust nature of Mo_2_C after 20 h of working operation, whereas W_2_C showed slight degradation. Hence, the observed deliverables established the capability of chemically reduced Mo_2_C catalysts with long-term durability in alkaline and acidic media, which makes them interchangeable for high-cost materials.

Furthermore, the stability of the Mo_2_C and W_2_C catalysts was investigated by XPS and FESEM analysis after continuous 20 h-HER operation in electrolytic solution (acidic medium); the results are shown in Figures S9, S10. The observed results also confirm no dramatic changes in XPS and FESEM data after continuous 20 h HER operation.

## Conclusions

Chemically reduced Mo_2_C and W_2_C produced using an economical reduction method were successfully employed as electrocatalysts for application in HER. The observed HER results revealed that Mo_2_C and W_2_C nanoparticles had low overpotentials ($\eta_{-10mAcm^{-2}}$ = −134 and −116 mV and −173 and −130 mV for Mo_2_C and W_2_C nanoparticles, respectively) with small Tafel slopes (83 and 65 mV.dec^−1^ and 86 and 100 mV.dec^−1^ for Mo_2_C and W_2_C nanoparticles, respectively) in a 0.5 M H_2_SO_4_ and in 1 M KOH electrolyte media. Both carbide catalysts showed strong stability in the alkaline and acidic media for over 20 h of operation. Thus, this work shows a viable way to synthesize nanostructured TMC-based electrocatalysts for hydrogen production.

## Experimental Section

### Materials and Methods

The commercial Mo_2_C and W_2_C chemicals of reagent grade were acquired from Sigma-Aldrich and were used without further purification. The following procedure was followed to prepare the chemically reduced Mo_2_C and W_2_C nanostructures (Hussain et al., 2019a). First, 2 g of commercial powder was well-disseminated in 100 mL of ethanol in a beaker to form a clear solution with the assistance of a room temperature stirring process. Subsequently, 50 mL of liquid ammonia solution was mixed with the black solution mixture, which was followed by a magnetic stirring at 85°C until the ethanol was completely evaporated from the mixture. The settled residue was then purified with de-ionized water and alcohol, sometimes using a centrifuge process, and the resultant material was placed in an oven at 60°C for 6 h. Finally, the collected black powders were placed in a quartz tube, and their temperature was raised with a heating rate of 5°C/min to attain 850°C with the support of a CH_4_/Ar/H_2_-mixture gas flow (50 sccm). The final chemically reduced Mo_2_C and W_2_C samples were kept in a vacuum desiccator for further analysis.

### Electrochemical Measurements

The electrocatalytic HER properties were examined in an acid medium (0.5 M H_2_SO_4_ solution) and a alkaline medium (1 M KOH) by using a typical three-electrode setup. A saturated calomel electrode (SCE) and a carbon rod were used as the reference and counter electrodes, respectively. For the preparation of a working electrode, a 10:80:10 weight ratio of poly (vinylidene fluoride), active materials (W_2_C and Mo_2_C), and carbon black was used with an N-methyl-2-pyrrolidone solvent. The resultant sample was coated onto NF and dried overnight at 90°C. Mo_2_C- and W_2_C-loaded NF was employed as the working electrode. The LSV was recorded using a scan rate of 10 mV s^−1^ at room temperature. All the LSV performances were completed using SCE and then referenced to a reversible hydrogen electrode (RHE) scale using the following equation: E (RHE) = E (SCE) + E° (SCE) + 0.059 pH. An electrochemical impedance spectroscopy (EIS) study was performed within the 0.1 Hz to 1 MHz frequency with a perturbation voltage of 10 mV.

### Characterization

Mo_2_C and W_2_C nanoparticles were studied using Raman spectroscopy (Renishaw Invia RE04, Ar laser−512 nm), FE-SEM (HITACHI S-4700), a Rigaku Ultima IV diffractometer with Cu-Kα radiation (0.154 nm), JEOL-2010F TEM with the help of Gatan DM software (version 3.0), PHI 5000 Versa Probe XPS, and a 3Flex surface characterization analyzer for nitrogen adsorption and desorption measurement at 77 K (Micromeritics, USA).

## Data Availability Statement

All datasets generated for this study are included in the article/Supplementary Material.

## Author Contributions

SH and JJ prepared the manuscript. SH performed the material synthesis. DV and AF actively took part in the characterization of catalysts. WS and K-SA performed XPS measurement and physical characterization of synthesized materials. H-SK, S-HC, and JJ did planning, design experimental work, and discussion. JJ edited the paper.

### Conflict of Interest

The authors declare that the research was conducted in the absence of any commercial or financial relationships that could be construed as a potential conflict of interest.
