# Supplementary material for: Synthesis of Mo2C and W2C Nanoparticle Electrocatalysts for the Efficient Hydrogen Evolution Reaction in Alkali and Acid Electrolytes
Source: Front Chem. 2019 Oct 25;7:716. doi: 10.3389/fchem.2019.00716 (PMC6823202; doi:10.3389/fchem.2019.00716)
Supplement: Supplementary file 1 [file Data_Sheet_1.docx]

**Synthesis of Mo_2_C and W_2_C nanoparticles electrocatalysts for the efficient hydrogen evolution reaction in alkali and acid electrolytes**

Sajjad Hussain^a, b^, Dhanasekaran Vikraman^c^, Asad Feroze^a,d^, Wooseok Song^e^, Ki-Seok An^e^, Hyun-Seok Kim^c^, Seung-Hyun Chun^a,d^, and Jongwan Jung^a,b*^

^a^Graphene Research Institute, Sejong University, Seoul - 05006, Republic of Korea.

^b^Department of Nano and Advanced Materials Engineering, Sejong University, Seoul -05006, Republic of Korea.

^c^Division of Electronics and Electrical Engineering, Dongguk University-Seoul, Seoul - 04620, Republic of Korea.

^d^Department of Physics, Sejong University, Seoul 05006, Republic of Korea.

^e^Thin Film Materials Research Center, Korea Research Institute of Chemical Technology, Daejeon 34114, Republic of Korea.

* Corresponding author’s E-mail: [jwjung@sejong.ac.kr](mailto:jwjung@sejong.ac.kr)


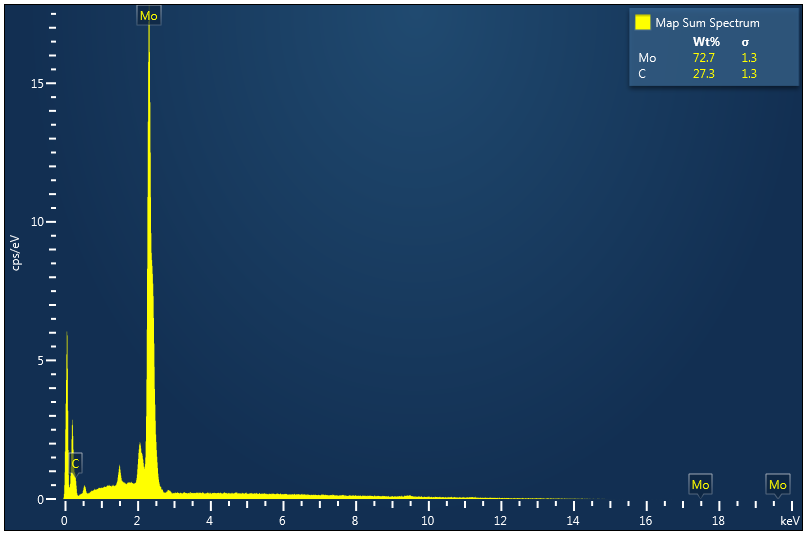


**Figure S1.** EDS pattern of chemically reduced Mo_2_C Nanoparticles.


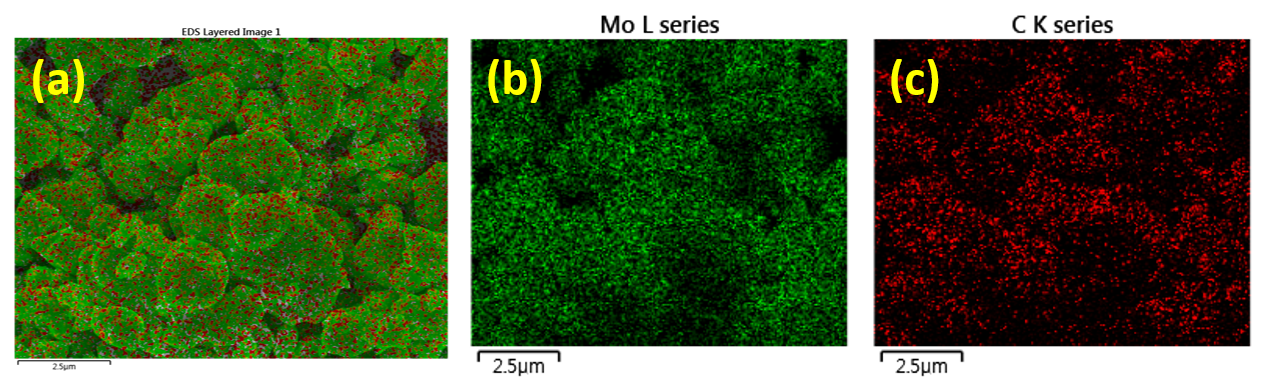


**Figure S2**. **(a-c)** FESEM EDS mapping and their element mapping images for chemically reduced Mo_2_C nanoparticles.


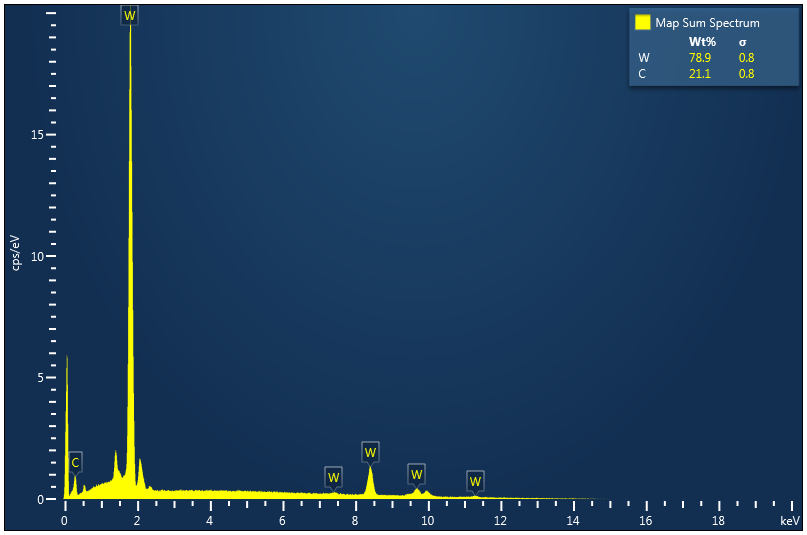


**Figure S3.** EDS pattern of chemically reduced W_2_C Nanoparticles.


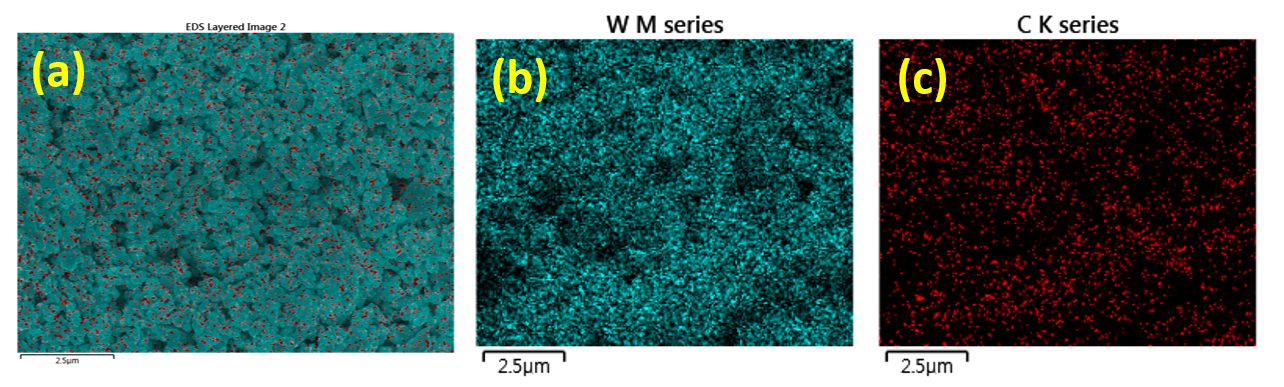


**Figure S4.** **(a-c)** FESEM EDS mapping and their element mapping images for chemically reduced W_2_C nanoparticles.


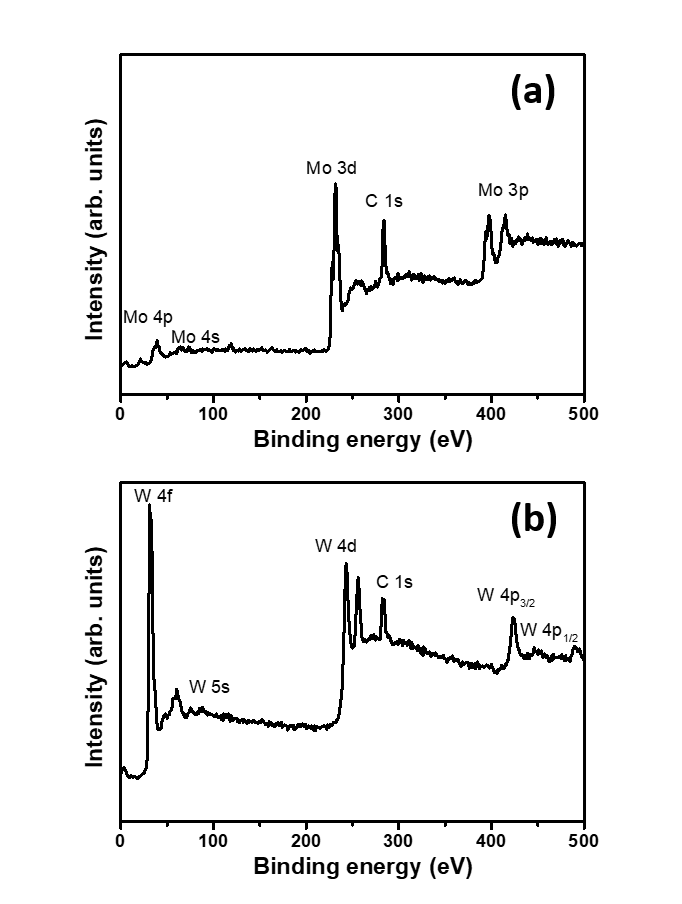


**Figure S5.** XPS survey scans for chemically reduced (a) Mo_2_C nanoparticles and (b) W_2_C nanoparticles.


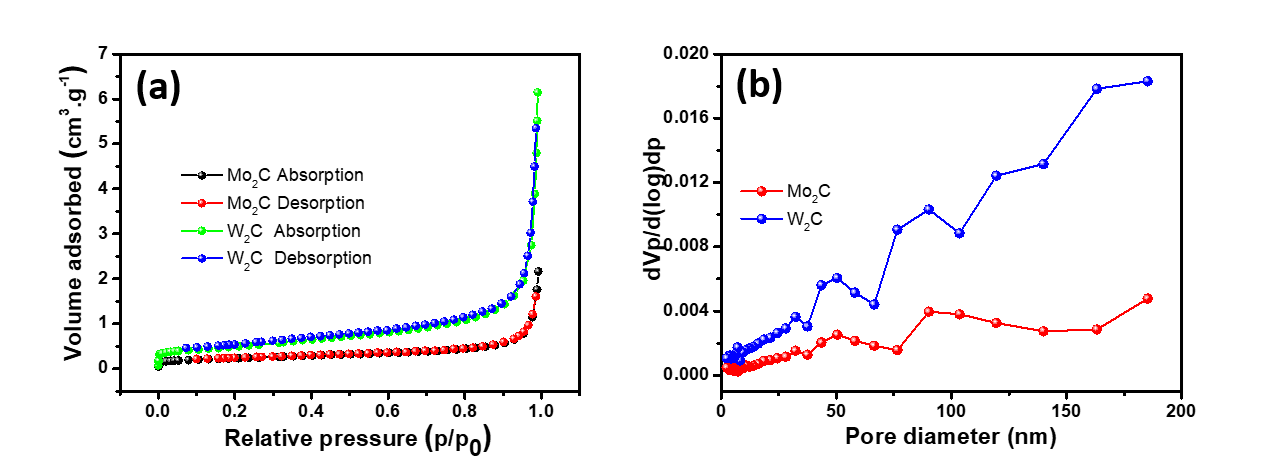


**Figure S6**. (a) Nitrogen adsorption-desorption isotherms and (b) pore diameter variations for chemically reduced Mo_2_C nanoparticles and W_2_C nanoparticles.


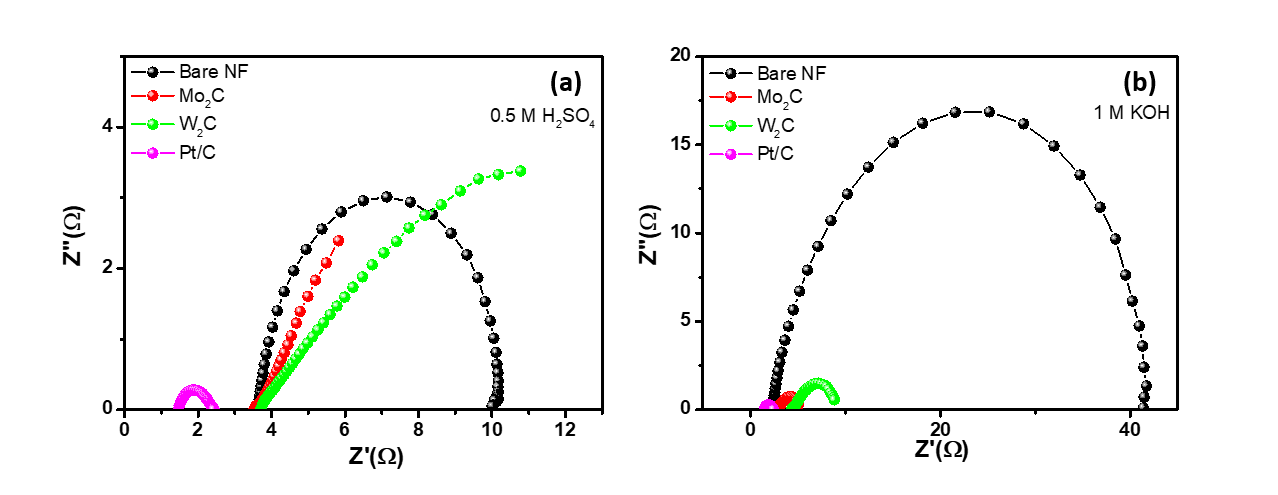


**Figure S7.** Nyquist plots of bare NF, Pt/C, Mo_2_C, and W_2_C in **(a)** acidic and **(b)** alkaline medium.


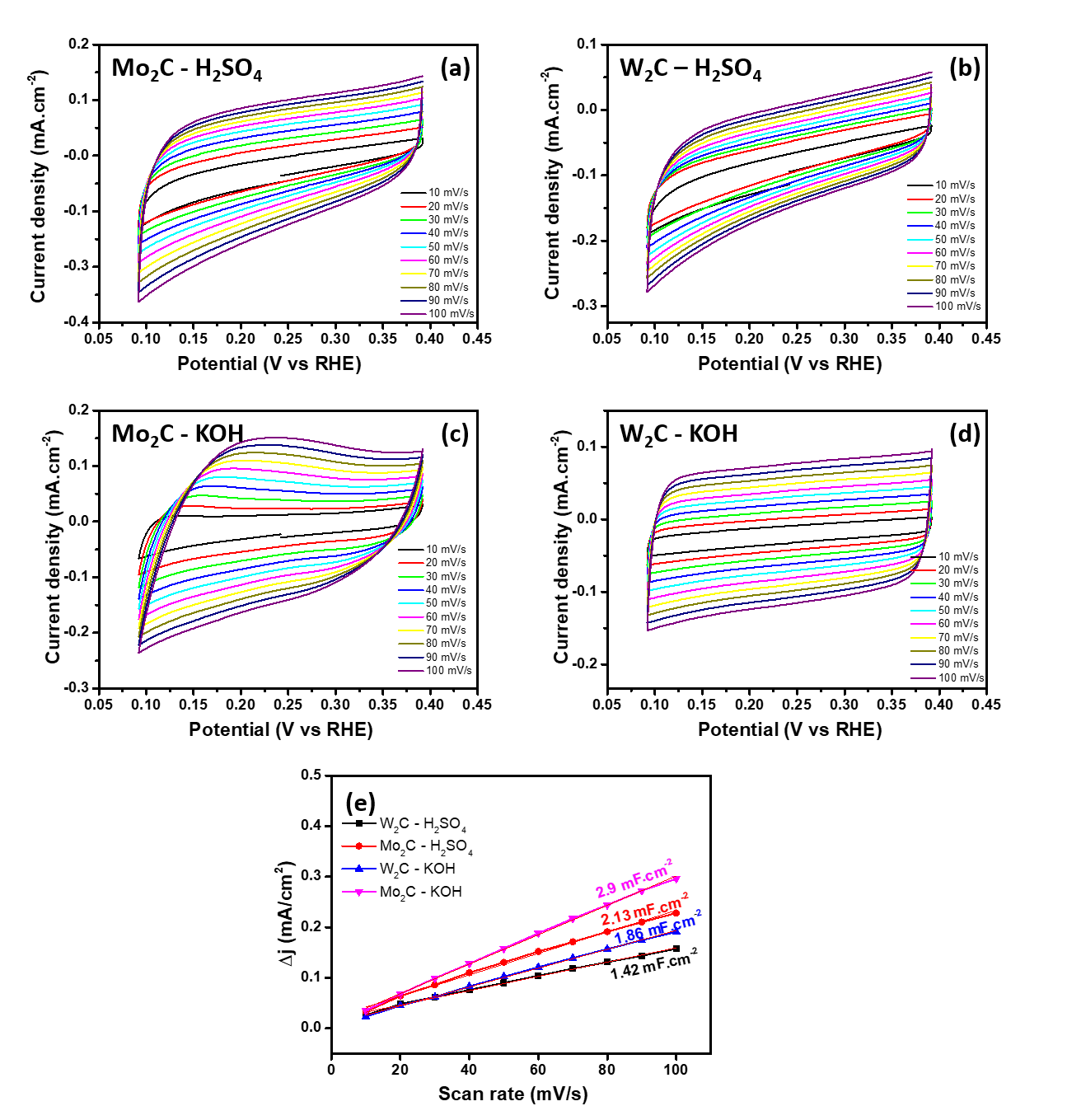


**Figure S8.** Electrochemical double-layer capacitance (Cdl) measurements. (a-d) Cyclic voltammograms of catalysts (a) Mo_2_C, and (b) W_2_C in 0.5M H_2_SO_4_ medium; (c) Mo_2_C, and (d) W_2_C in 1M KOH medium. (e) Linear fitting of the capacitive current differences (at 0.24 V vs RHE) of the catalysts versus the scan rates.


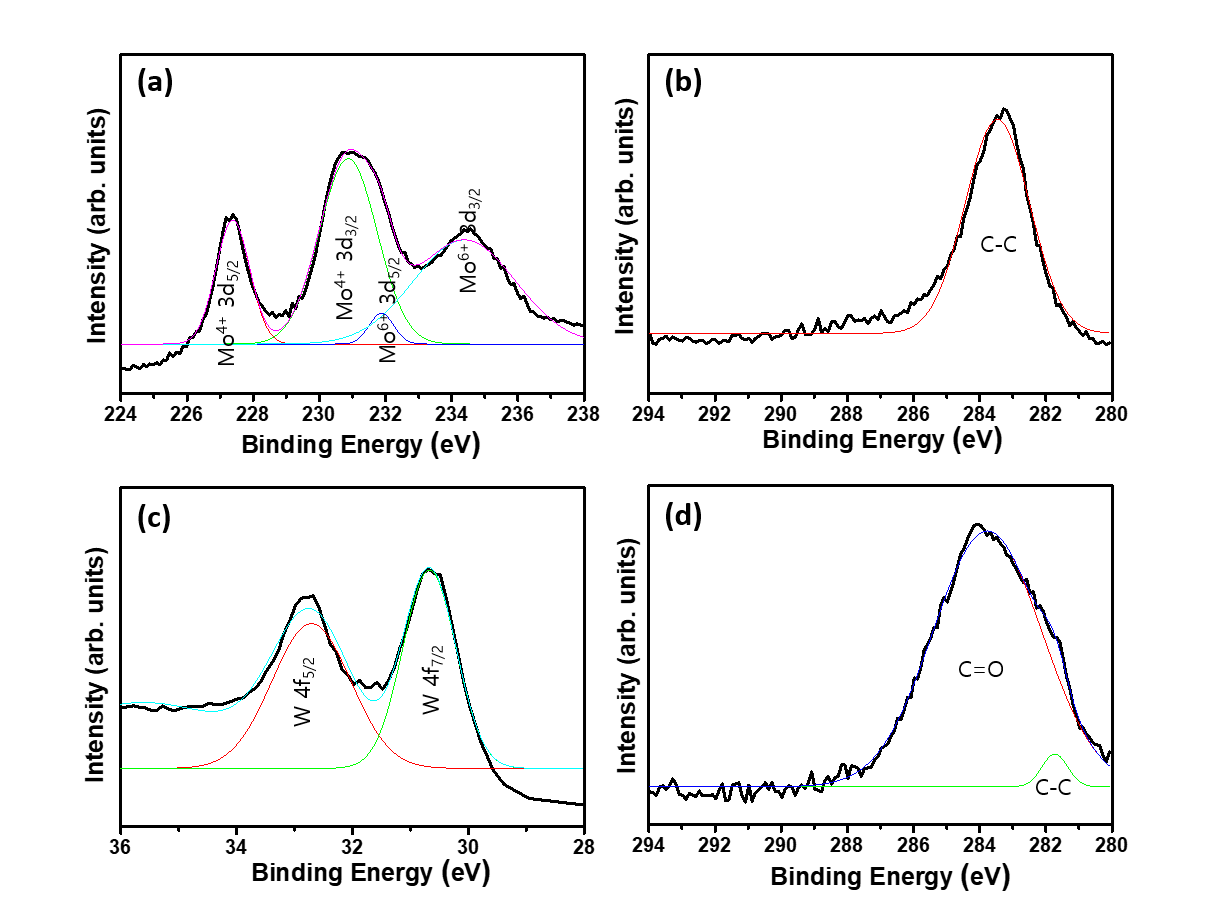


**Figure S9.** XPS spectra for stability after 20 h HER performance: (a) Mo 3d, (b) C 1s binding energy for Mo_2_C; (c) W 4f, and (d) C 1s binding energy for W_2_C.


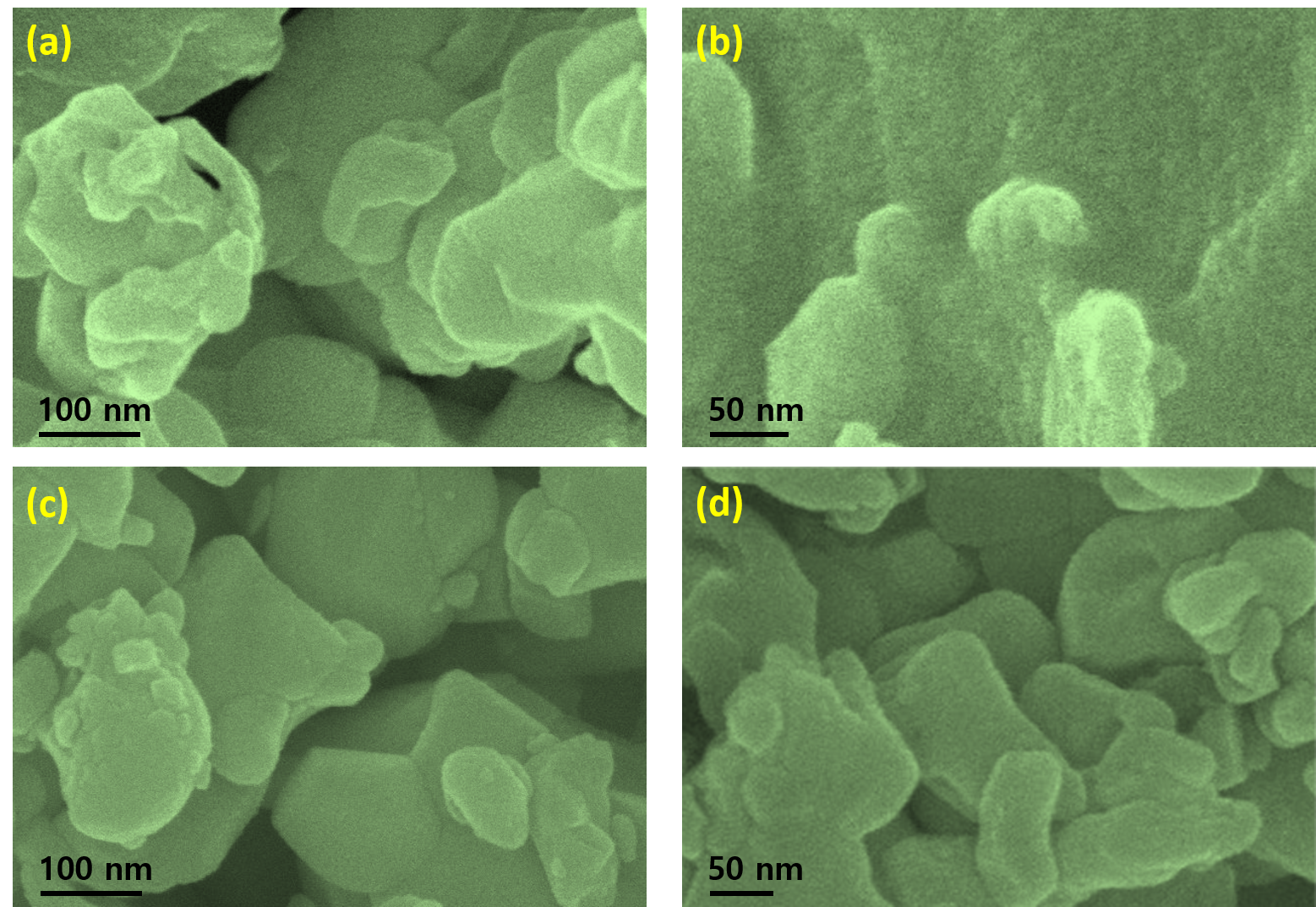


**Figure S10.** Stability confirmation by FESEM after 20 h HER performance: **(a-b)** Mo_2_C and **(c-d)** W_2_C electrocatalysts.

**Table S1.** HER catalytic performances Mo_2_C-based electrocatalysts.

| **Electrocatalyst** | **Electrolyte** | **η (mV)** | **Tafel Slope (mV·dec^-1^)** | **j_0_**  **(mA·cm^-2^)** | **Ref** |
| --- | --- | --- | --- | --- | --- |
| **Mo_2_C nanoparticles** | **0.5 M H_2_SO_4_** | **-134 @ 10 mA/cm^2^** | **83** | **0.131** | **This work** |
| **Mo_2_C nanoparticles** | **1.0 M KOH** | **-116 @ 10 mA/cm^2^** | **65** | **0.846** | **This work** |
| Mo_2_C@NPC/NPRGO | 0.5 M H_2_SO_4_ | -34@ 10 mA/cm^2^ | 33.6 | 1.09 | [^1^](#_ENREF_1) |
| Mo_2_C@N‐doped carbon (NC) | 0.5 M H_2_SO_4_ | -124 @ 10 mA/cm^2^ | 60 | 9.6×10^-2^ | [^2^](#_ENREF_2) |
| Reduced GO-Mo_2_C composites | 0.5 M H_2_SO_4_ | -206 @ 10 mA/cm^2^ | 67 | 125 | [^3^](#_ENREF_3) |
| Mo_2_C/mesoporous carbon | 0.1 M KOH | -165 @ 10 mA/cm^2^ | 63.3 | - | [^4^](#_ENREF_4) |
| MoC-Mo_2_C  Heteronanowires | 0.5 M H_2_SO_4_ and 1.0 M KOH | -126 & -120@ 10 mA/cm^2^ | 43 & 42 | 1.1×10^-2^ | [^5^](#_ENREF_5) |
| β-Mo_2_C Nanotubes | 0.5 M H_2_SO_4_ and 1.0 M KOH | -172 & -112@ 10 mA/cm^2^ | 62 & 55 | 0.017 & 0.087 | [^6^](#_ENREF_6) |
| β-Mo_2_C nanoparticles | 0.5 M H_2_SO_4_ | -60@ 200 mA/cm^2^ | 55 |  | [^7^](#_ENREF_7) |
| Mo_2_C/CNT | 1.0 M HClO_4_ solution | -64@ 1 mA/cm^2^ | 52.2 | 1.4×10^-2^ | [^8^](#_ENREF_8) |
| MoO_2_/α-Mo2C heterojunction | 0.5 M H_2_SO_4_ and 1.0 M KOH | -152 & -100@ 10 mA/cm^2^ | 65 & 50 | 4.42×10^-2^ | [^9^](#_ENREF_9) |
| Mo_2_C Nanoparticles | 0.5 M H_2_SO_4_ and 1.0 M KOH | -180 & -210@ 10 mA/cm^2^ | 49 & 48 | 3×10^-3^ | [^10^](#_ENREF_10) |
| Mo_2_C/CC | 0.5 M H_2_SO_4_ | -140@ 10 mA/cm^2^ | 124 |  | [^11^](#_ENREF_11) |
| Mo_2_C/ N doped  carbon nanotubes | 0.5 M H_2_SO_4_ | -147@ 10 mA/cm^2^ | 71 | 72.7@200 mV | [^12^](#_ENREF_12) |
| Mo_2_C Nanoparticles/ Graphitic CC | 0.5 M H_2_SO_4_ | -200@ 10 mA/cm^2^ | 62.6 | 12.5×10^-3^ | [^13^](#_ENREF_13) |
| Mo_2_C Nanoparticles/ N doped porous  carbon nanofibers | 0.5 M H_2_SO_4_ and 1.0 M KOH | -85 & -90@ 1 mA/cm^2^ | 68 & 60.2 | 0.178 | [^14^](#_ENREF_14) |
| Mo_2_C nanoparticles | 0.5 M H_2_SO_4_ and 1.0 M KOH | -144 & -100@ 10 mA/cm^2^ | 55 & 65 | - | [^15^](#_ENREF_15) |
| Mo_2_C QDs/ N-doped graphitic carbon layers | 0.5 M H_2_SO_4_ and 1.0 M KOH | -136 & -111@ 10 mA/cm^2^ | 68 & 57.8 | - | [^16^](#_ENREF_16) |
| Mo_2_C/CNT-graphene | 0.5 M H_2_SO_4_ | -130@ 10 mA/cm^2^ | 58 | 6.20×10^-2^ | [^17^](#_ENREF_17) |
| Mo_2_C/Graphene Nanoribbons | 0.5 M H_2_SO_4_ and 1.0 M KOH | -167 & -217@ 10 mA/cm^2^ | 63 & 64 | - | [^18^](#_ENREF_18) |
| α-Mo2C | 0.5 M H_2_SO_4_ and 1.0 M KOH | -198 & -176@ 10 mA/cm^2^ | 56 &58 | - | [^19^](#_ENREF_19) |
| MoS_2_/Ti3C_2_ | 0.5 M H_2_SO_4_ | -110@ 10 mA/cm^2^ | 40 | - | [^20^](#_ENREF_20) |
| Hierarchical Mo_2_C/C | 0.5 M H_2_SO_4_ and 1.0 M KOH | -180 & -125@ 10 mA/cm^2^ | 72 &125 | - | [^21^](#_ENREF_21) |
| Mo_2_C NPs | 0.5 M H_2_SO_4_ | -78@ 10 mA/cm^2^ | 41 | 0.179 | [^22^](#_ENREF_22) |
| Porous Mo_2_C  architecture | 0.5 M H_2_SO_4_ and 1.0 M KOH | -166 & -139@ 10 mA/cm^2^ | 71 &75 | 0.441 & 0.287 | [^23^](#_ENREF_23) |
| Mo_2_C nanostructures | 0.5 M H_2_SO_4_ | -160@ 10 mA/cm^2^ | 64.5 |  | [^24^](#_ENREF_24) |
| rich N-doped Mo2C | 0.5 M H_2_SO_4_ | -150@ 10 mA/cm^2^ | 61 | 10 | [^25^](#_ENREF_25) |
| MoO_2_@PC-RGO | 0.5 M H_2_SO_4_ | -64@ 10 mA/cm^2^ | 41 | 4.8 × 10^-4^ | [^26^](#_ENREF_26) |
| MoCx nano- octahedrons | 0.5 M H_2_SO_4_ and 1.0 M KOH | -142 & -151@ 10 mA/cm^2^ | 53 & 59 | 0.023 & 0.029 | [^27^](#_ENREF_27) |
| P-Mo_2_C@C nanowires | 0.5 M H_2_SO_4_ | -89 @ 10 mA/cm^2^ | 42 | 1.8 × 10^-1^ | [^28^](#_ENREF_28) |
| W_4_MoC | 0.5 M H_2_SO_4_ | -184 @ 80 mA/cm^2^ | 52 | 2.9 × 10^-2^ | [^29^](#_ENREF_29) |
| Porous N@MoPC | 0.5 M H_2_SO_4_ | -108 @ 10 mA/cm^2^ | 69.4 | 0.3424 | [^30^](#_ENREF_30) |
| SWCNTs/MoSe_2_ | 0.5 M H_2_SO_4_ | 100 @ 10 mA/cm^2^ | 63 | 0.203 | [^31^](#_ENREF_31) |
| MoSe_2_/Bi_2_Se_3_ hybrids | 0.5 M H_2_SO_4_ | 300 mV @ 85 mA/cm^2^ | 44 | - | [^32^](#_ENREF_32) |
| MoSe_2_@MoS_2_ | 0.5 M H_2_SO_4_ | 161 @ 10 mA/cm^2^ | 60 | - | [^33^](#_ENREF_33) |
| MoSe_2_/NiSe_2_ composite nanowires | 0.5 M H_2_SO_4_ | 249 @ 100 mA/cm^2^ | 46.9 | - | [^34^](#_ENREF_34) |
| NiS_2_/MoS_2_ | 1.0 M KOH | 102 @ 10 mA/cm^2^ | - | 53.7 | [^35^](#_ENREF_35) |

**Table S2.** HER catalytic performances W_2_C-based electrocatalysts.

| **Electrocatalyst** | **Electrolyte** | **η (mV)** | **Tafel Slope (mV·dec^-1^)** | **j_0_**  **(mA·cm^-2^)** | **Ref** |
| --- | --- | --- | --- | --- | --- |
| **W_2_C nanoparticles** | **0.5 M H_2_SO_4_** | **-173 @ 10 mA/cm^2^** | **86** | **0.194** | **This work** |
| **W_2_C nanoparticles** | **1.0 M KOH** | **-130 @ 10 mA/cm^2^** | **100** | **0.438** | **This work** |
| P-modified W_2_C/ N-doped carbon (P-W_2_C@NC) | 0.5 M H_2_SO_4_ | -89@ 10 mA/cm^2^ | 53 | 0.316 | [^36^](#_ENREF_36) |
| WC-CNTs | 0.5 M H_2_SO_4_ and 1.0 M KOH | -145 & -106 @ 10 mA/cm^2^ | 72 & 106 | 300 mV of 117.6 & 33.1 mV | [^37^](#_ENREF_37) |
| WC/W_2_C@C NWs | 0.5 M H_2_SO_4_ and 1.0 M KOH | -69 & -56 @ 10 mA/cm^2^ | 56& 59 | 0.361 | [^38^](#_ENREF_38) |
| W_2_C nanoparticles | 0.5 M H_2_SO_4_ | -123@ 10 mA/cm^2^ | 45 | - | [^39^](#_ENREF_39) |
| WC nanoparticles | 0.5 M H_2_SO_4_ | -51@ 10 mA/cm^2^ | 49 | 2.4 | [^40^](#_ENREF_40) |
| WC/W phosphide-N-doped carbon (W_2_C/WP@NC) | 0.5 M H_2_SO_4_ | -83@ 10 mA/cm^2^ | 61 | 0.313 | [^41^](#_ENREF_41) |
| WOx nanowires | 0.5 M H_2_SO_4_ | -108@ 10 mA/cm^2^ | 46 | 0.318 | [^42^](#_ENREF_42) |
| TC nanowalls | 0.5 M H_2_SO_4_ | -160@ 10 mA/cm^2^ | 67 |  | [^43^](#_ENREF_43) |
| Phosphorus-modified  (P-WN/rGO) | 0.5 M H_2_SO_4_ | -85@ 10 mA/cm^2^ | 54 | 0.35 | [^44^](#_ENREF_44) |
| WC-graphene heterostructures | 0.5 M H_2_SO_4_ | -120@ 10 mA/cm^2^ | 38 | - | [^45^](#_ENREF_45) |
| WO_3-x_ Nanoplates/CNF | 0.5 M H_2_SO_4_ | -185@ 10 mA/cm^2^ | 89 | 0.239 | [^46^](#_ENREF_46) |

**References**

1. Li, J.-S.; Wang, Y.; Liu, C.-H.; Li, S.-L.; Wang, Y.-G.; Dong, L.-Z.; Dai, Z.-H.; Li, Y.-F.; Lan, Y.-Q. *Nature communications* **2016,** 7, 11204.

2. Liu, Y.; Yu, G.; Li, G. D.; Sun, Y.; Asefa, T.; Chen, W.; Zou, X. *Angewandte Chemie International Edition* **2015,** 54, (37), 10752-10757.

3. Ojha, K.; Saha, S.; Kolev, H.; Kumar, B.; Ganguli, A. K. *Electrochimica Acta* **2016,** 193, 268-274.

4. Qamar, M.; Adam, A.; Merzougui, B.; Helal, A.; Abdulhamid, O.; Siddiqui, M. *Journal of Materials Chemistry A* **2016,** 4, (41), 16225-16232.

5. Lin, H.; Shi, Z.; He, S.; Yu, X.; Wang, S.; Gao, Q.; Tang, Y. *Chemical science* **2016,** 7, (5), 3399-3405.

6. Ma, F. X.; Wu, H. B.; Xia, B. Y.; Xu, C. Y.; Lou, X. W. *Angewandte Chemie International Edition* **2015,** 54, (51), 15395-15399.

7. Tang, C.; Sun, A.; Xu, Y.; Wu, Z.; Wang, D. *Journal of Power Sources* **2015,** 296, 18-22.

8. Chen, W.-F.; Wang, C.-H.; Sasaki, K.; Marinkovic, N.; Xu, W.; Muckerman, J.; Zhu, Y.; Adzic, R. *Energy & Environmental Science* **2013,** 6, (3), 943-951.

9. Liu, Y.; Huang, B.; Xie, Z. *Applied Surface Science* **2018,** 427, 693-701.

10. Wang, D.; Wang, J.; Luo, X.; Wu, Z.; Ye, L. *ACS Sustainable Chemistry & Engineering* **2017,** 6, (1), 983-990.

11. Fan, M.; Chen, H.; Wu, Y.; Feng, L.-L.; Liu, Y.; Li, G.-D.; Zou, X. *Journal of Materials Chemistry A* **2015,** 3, (31), 16320-16326.

12. Zhang, K.; Zhao, Y.; Fu, D.; Chen, Y. *Journal of Materials Chemistry A* **2015,** 3, (11), 5783-5788.

13. Cui, W.; Cheng, N.; Liu, Q.; Ge, C.; Asiri, A. M.; Sun, X. *ACS Catalysis* **2014,** 4, (8), 2658-2661.

14. Wang, H.; Sun, C.; Cao, Y.; Zhu, J.; Chen, Y.; Guo, J.; Zhao, J.; Sun, Y.; Zou, G. *Carbon* **2017,** 114, 628-634.

15. Huang, Y.; Gong, Q.; Song, X.; Feng, K.; Nie, K.; Zhao, F.; Wang, Y.; Zeng, M.; Zhong, J.; Li, Y. *ACS nano* **2016,** 10, (12), 11337-11343.

16. Pu, Z.; Wang, M.; Kou, Z.; Amiinu, I. S.; Mu, S. *Chemical Communications* **2016,** 52, (86), 12753-12756.

17. Youn, D. H.; Han, S.; Kim, J. Y.; Kim, J. Y.; Park, H.; Choi, S. H.; Lee, J. S. *ACS nano* **2014,** 8, (5), 5164-5173.

18. Gao, W.; Shi, Y.; Zhang, Y.; Zuo, L.; Lu, H.; Huang, Y.; Fan, W.; Liu, T. *ACS Sustainable Chemistry & Engineering* **2016,** 4, (12), 6313-6321.

19. Ma, L.; Ting, L. R. L.; Molinari, V.; Giordano, C.; Yeo, B. S. *Journal of Materials Chemistry A* **2015,** 3, (16), 8361-8368.

20. Attanayake, N. H.; Abeyweera, S. C.; Thenuwara, A. C.; Anasori, B.; Gogotsi, Y.; Sun, Y.; Strongin, D. R. *Journal of Materials Chemistry A* **2018,** 6, (35), 16882-16889.

21. Wu, C.; Li, J. *ACS applied materials & interfaces* **2017,** 9, (47), 41314-41322.

22. Ma, R.; Zhou, Y.; Chen, Y.; Li, P.; Liu, Q.; Wang, J. *Angewandte Chemie International Edition* **2015,** 54, (49), 14723-14727.

23. Meng, T.; Zheng, L.; Qin, J.; Zhao, D.; Cao, M. *Journal of Materials Chemistry A* **2017,** 5, (38), 20228-20238.

24. Ge, C.; Jiang, P.; Cui, W.; Pu, Z.; Xing, Z.; Asiri, A. M.; Obaid, A. Y.; Sun, X.; Tian, J. *Electrochimica Acta* **2014,** 134, 182-186.

25. Chi, J.-Q.; Yan, K.-L.; Gao, W.-K.; Dong, B.; Shang, X.; Liu, Y.-R.; Li, X.; Chai, Y.-M.; Liu, C.-G. *Journal of Alloys and Compounds* **2017,** 714, 26-34.

26. Tang, Y. J.; Gao, M. R.; Liu, C. H.; Li, S. L.; Jiang, H. L.; Lan, Y. Q.; Han, M.; Yu, S. H. *Angewandte Chemie International Edition* **2015,** 54, (44), 12928-12932.

27. Wu, H. B.; Xia, B. Y.; Yu, L.; Yu, X.-Y.; Lou, X. W. D. *Nature communications* **2015,** 6, 6512.

28. Shi, Z.; Nie, K.; Shao, Z.-J.; Gao, B.; Lin, H.; Zhang, H.; Liu, B.; Wang, Y.; Zhang, Y.; Sun, X. *Energy & Environmental Science* **2017,** 10, (5), 1262-1271.

29. Xiao, P.; Ge, X.; Wang, H.; Liu, Z.; Fisher, A.; Wang, X. *Advanced Functional Materials* **2015,** 25, (10), 1520-1526.

30. Huang, Y.; Ge, J.; Hu, J.; Zhang, J.; Hao, J.; Wei, Y. *Advanced Energy Materials* **2018,** 8, (6), 1701601.

31. Leyla, N.; Sebastiano, B.; Reinier, O.-N.; Alberto, A.; Mirko, P.; Esau, D. R. C. A.; Francesco, B. *Adv. Energy Mater.* **2018,** 8, (16), 1703212.

32. Yang, J.; Wang, C.; Ju, H.; Sun, Y.; Xing, S.; Zhu, J.; Yang, Q. *Adv. Funct. Mater.* **2017,** 27, (48), 1703864.

33. Ren, X.; Wei, Q.; Ren, P.; Wang, Y.; Chen, R. *Mater. Lett.* **2018,** 231, 213-216.

34. Zhang, L.; Wang, T.; Sun, L.; Sun, Y.; Hu, T.; Xu, K.; Ma, F. *J. Mater. Chem. A* **2017,** 5, (37), 19752-19759.

35. Wang, J.; Liu, Z.; Zhan, C.; Zhang, K.; Lai, X.; Tu, J.; Cao, Y. *J. Mater. Sci. Technol.* **2019**.

36. Yan, G.; Wu, C.; Tan, H.; Feng, X.; Yan, L.; Zang, H.; Li, Y. *Journal of Materials Chemistry A* **2017,** 5, (2), 765-772.

37. Fan, X.; Zhou, H.; Guo, X. *ACS nano* **2015,** 9, (5), 5125-5134.

38. Zhang, L.-N.; Ma, Y.-Y.; Lang, Z.-L.; Wang, Y.-H.; Khan, S. U.; Yan, G.; Tan, H.-Q.; Zang, H.-Y.; Li, Y.-g. *Journal of Materials Chemistry A* **2018,** 6, (31), 15395-15403.

39. Gong, Q.; Wang, Y.; Hu, Q.; Zhou, J.; Feng, R.; Duchesne, P. N.; Zhang, P.; Chen, F.; Han, N.; Li, Y. *Nature communications* **2016,** 7, 13216.

40. Xu, Y.-T.; Xiao, X.; Ye, Z.-M.; Zhao, S.; Shen, R.; He, C.-T.; Zhang, J.-P.; Li, Y.; Chen, X.-M. *Journal of the American Chemical Society* **2017,** 139, (15), 5285-5288.

41. Shi, M.; Li, W.; Fang, J.; Jiang, Z.; Gao, J.; Chen, Z.; Sun, F.; Xu, Y. *Electrochimica Acta* **2018,** 283, 834-841.

42. Liu, C.; Qiu, Y.; Xia, Y.; Wang, F.; Liu, X.; Sun, X.; Liang, Q.; Chen, Z. *Nanotechnology* **2017,** 28, (44), 445403.

43. Ko, Y.-J.; Cho, J.-M.; Kim, I.; Jeong, D. S.; Lee, K.-S.; Park, J.-K.; Baik, Y.-J.; Choi, H.-J.; Lee, W.-S. *Applied Catalysis B: Environmental* **2017,** 203, 684-691.

44. Yan, H.; Tian, C.; Wang, L.; Wu, A.; Meng, M.; Zhao, L.; Fu, H. *Angewandte Chemie International Edition* **2015,** 54, (21), 6325-6329.

45. Zeng, M.; Chen, Y.; Li, J.; Xue, H.; Mendes, R. G.; Liu, J.; Zhang, T.; Ruemmeli, M. H.; Fu, L. *Nano Energy* **2017,** 33, 356-362.

46. Chen, J.; Yu, D.; Liao, W.; Zheng, M.; Xiao, L.; Zhu, H.; Zhang, M.; Du, M.; Yao, J. *ACS applied materials & interfaces* **2016,** 8, (28), 18132-18139.
